# The Effect of Unilateral Spinal Anaesthesia and Psoas Compartment with Sciatic Block on the Postoperative Pain Management in Total Knee Artroplastic Surgery

**DOI:** 10.1155/2017/4127424

**Published:** 2017-01-31

**Authors:** Ebru Canakci, Dogus Unal, Yunus Guzel

**Affiliations:** ^1^Department of Anesthesiology, Ordu University, School of Medicine, Ordu, Turkey; ^2^Department of Anesthesiology, Ordu State Hospital, Ordu, Turkey; ^3^Department of Orthopedics and Traumatology, Ordu University, School of Medicine, Ordu, Turkey

## Abstract

*Purpose*. This study was designed to investigate the effects of peripheral nerve block methods, applied through unilateral spinal anaesthesia on elderly patients to undergo total knee arthroplasty, on perioperative hemodynamic parameters and postoperative analgesia period.* Materials and Method*. 60 patients were randomly divided into two groups in the study. In group USA spinal anaesthesia was performed. In group PCS it was applied on psoas compartment block and sciatic nerve block.* Results*. Significantly higher intraoperative 60th and 90th minute mean arterial pressure values were ascertained in the PCS group compared to the USA group. The decrease observed in the 5th, 10th, and 20th minute MAP values in the USA group was statistically significant according to the control MAP value. Concerning within group comparisons, the decrease in 5th, 10th, and 20th minute heart rate values in the USA group was statistically significant compared with the control measurement value. The mean beginning time of sensory and motor blocks in the PCS group was found to be at a significantly advanced level compared with that in the USA group.* Conclusions*. The PCS block technique using bupivacaine hydrochloride ensured a higher haemodynamic efficiency in the perioperative period in high-risk elderly patients undergoing total knee arthroplasty. This trial is registered with ClinicalTrials.gov Identifier: NCT03021421.

## 1. Introduction

Majority of patients who undergo orthopaedic lower extremity surgery (OLES) such as total knee arthroplasty are in the advanced age group. Presence of cardiac, endocrine, renal, cerebral, and respiratory tract diseases increases morbidity risk during and after operations among these patients [1]. In addition, postoperative pain treatment of these patients also poses a problem for anaesthetists [2]. Anaesthesia approach for these patients generally includes general anaesthesia (GA), central neuraxial block, and usage of systematic analgesic for postoperative pain treatment. Psoas compartment block (PCB) is an alternative approach used to overcome many side effects related to GA and central neuraxial block techniques. Sciatic nerve block combined with psoas compartment block ensures unilateral lower extremity anaesthesia. Recently, sciatic nerve block combined with psoas compartment block is used as an alternative technique to central neuraxial block and GA. Femoral, lateral femoral cutaneous, and obturator nerves are simultaneously blocked with psoas compartment block [3].

The purpose of this study was to compare the USA and PCB and psoas compartment-sciatic (PCS) techniques to be used in the application of anaesthesia in patients undergoing total knee arthroplasty, as well as their effects on anaesthesia, the surgical preparation periods, and the haemodynamic parameters.

## 2. Materials and Methods

After obtaining the approval from the Ethics Committee of Samsun Ondokuz Mayis University (date: 01/15/2015; decision number B.30.2.ODM.0.20.08/1642) and informed consent from the participants, 60 volunteer patients (44 females, 16 males) who were planned for total knee arthroplasty intervention using the regional anaesthesia method were classified as ASA II-III, were aged between 50 and 80 years, and were included in the study. In this prospective, randomised, multicentric, and single-blind study, 60 patients who were diagnosed with gonarthrosis in the outpatient clinic of Ordu University Training Hospital and Ordu State Hospital between January 1, 2015, and June 30, 2015, and planned for total knee arthroplasty were included. Exclusion criteria were patients who were allergic to local anaesthetics, had neurological disease, had infections in the intervention area, did not cooperate, or did not agree to the intervention. After receiving informed consent from the patients, they were divided into two groups using the closed envelope method: the USA group was the spinal anaesthesia (SA) group (*n* = 30), and the PCS group was the PCB and sciatic nerve block group (*n* = 30). All of the patients planned for surgery were monitored in terms of noninvasive arterial blood pressure, heart rate (HR), and peripheral haemoglobin oxygen saturation (SpO_2_). Peripheral vascular access was established using an 18-G cannula before the block operation, and 500 mL of 0.9% NaCl solution was infused for 20 minutes followed by the intravenous administration of 0.07 mg/kg midazolam (Dormicum®; Roche, Basel, Switzerland). The haemodynamic parameters were recorded every 5 minutes until the end of the preoperative and perioperative period. Next, 2 mL of hyperbaric bupivacaine hydrochloride (Marcain Heavy 0.5%; AstraZeneca®, London, UK) was given to patients in the USA group in the lateral decubitus position, after regional sterilisation, through the selected intervertebral space (randomly L4-L5 or L3-L4 segment) using a 25-G Quincke spinal injector (B. Braun®, Melsungen, Germany). After remaining in this position for 15 minutes following the procedure, the patients were changed to the supine position. Patients in the PCS group were changed to the lateral decubitus (Simms) position. Following the sterilisation and covering procedures required for blockade of the lumbar plexus somatic nerves, the abdominal settings were selected on the ultrasound device (Mindray DC-T6 VET®, Shenzhen, China). The depth was set at 11-12 cm, and the frequency was set at 4–8 MHz. A convex ultrasonography (USG) probe was placed 4 cm laterally on the middle line at the L3–L5 level. The transverse process was longitudinally identified using an USG probe. The “trident sign” (three-pronged spear appearance) acoustic shadow of the transverse process is specific for this peripheric nerve block (=psoas compartment block). After viewing the psoas muscle at the depth of the transverse process, the needle was inserted using an out-plane approach. While using a nerve stimulator simultaneously (Stimupleks A; B. Braun®, Melsungen AG, Germany), the stimulation current was reduced to the 0.2–0.5 mA level when contraction of the quadriceps muscle was observed, and 20 mL of local anaesthetic (5 mL of 2% lidocaine + 15 mL of 0.5% bupivacaine) was injected. The local anaesthetic was observed to disperse around the lumbar plexus (femoral nerve, lateral femoral cutaneous nerve, obturator nerve, and posterior femoral cutaneous nerve) with USG. Sciatic nerve block was applied as follows: the patients' positions were not changed, and the skin was disinfected in the same position (lateral decubitus position). Following the required covering process, the USG settings were applied for optimum visualisation. The depth was set as 3–6 cm and frequency as 2–8 MHz. The convex USG probe was placed in the subgluteal area in the transverse position. After determining the sciatic nerve, the injector was inserted into the lateral edge of the probe as in-plane and proceeded towards the nerve. When using the nerve stimulator simultaneously (Stimupleks A; B. Braun®), the stimulation current was reduced to 0.2–0.5 mA until dorsiflexion motion on foot became visible. The local anaesthetic was observed to disperse around the sciatic nerve with USG by administering 20 mL of local anaesthetic (5 mL of 2% lidocaine + 15 mL of 0.5% bupivacaine) following negative aspiration with the injector. The patients were changed to the supine position. Surgery was allowed when effective sensory block reached with at least a T10 dermatome.

The application times of both techniques were recorded as the technique application times. While the analgesia level was evaluated using the pinprick test, the degree of motor block was assessed using the modified Bromage scale (0: no block, 1: hip flexion is blocked when the knee is extended, 2: knee flexion is blocked, and 3: full motor block). Additionally, the sensory and motor blocks were evaluated at 5-minute intervals using the measurements of the haemodynamic parameters after the administration of medications. No case was excluded from the study because of block failure.

Surgical intervention was initiated after the development of a full motor block. The time from the beginning of surgical incision until the completion of the procedure was recorded as the operation period. All of the patients were given 3 L/minute oxygen through the mask and 5 mL/kg/hour maintenance crystalloid i.v. fluid during the surgery. A decrease in the mean basal arterial pressure of 25% or more was considered as hypotension; in this case, ephedrine was given as 5 mg intravenously (i.v.), and 5 mL/kg additional colloid fluid was given. Heart rates less than 50 beats/minute were considered as bradycardia, and atropine was given as 0.5 mg i.v. The motor block return time and first analgesic requirement time were recorded. Pain control was assessed by VAS score. If the VAS score was above 40 mm, 1 mL/kg tramadol hcl (Ultramex® 100 mg ampule, Adeka, Istanbul, Turkey) was given intravenously.

### 2.1. Statistical Analysis

The data obtained from the study were evaluated using the SPSS for Windows software (ver. 21.0; SPSS Inc., Chicago, IL, USA). The Kolmogorov-Smirnov distribution test was used to analyse the normal distribution. Pearson's chi-squared test was used to compare qualitative data (gender, ASA status, block success percentage, VAS score, satisfaction scores, and complication evaluations). If there were two groups in the comparison of quantitative data (age, weight, height, operation time, MAP, HR, sensory and motor block beginning times, surgery beginning time, sensory and motor block periods, and time of first analgesic need), the Mann–Whitney *U*-test was used for between-group comparisons of the parameters. The Wilcoxon signed-rank test was used for within-group comparisons of the parameters. Data are provided as means ± standard deviation (and as median values). The number of samples of a study with high evidence power must be high; for this reason, power analysis was done. Considering the study of Demirel et al. as the basis on hemodynamic stability for this study, according to the power analysis and sample size test made at *α* = 0.05 and 80% power, the sample size for each group was defined as 25. In the current study, 30 patients were included in each group. The *p* value was defined as less than 0.05 for significant differences and less than 0.01 for advanced significant differences.

## 3. Results

In total, 60 patients, including 44 females (73.3%) and 16 males (26.6%) aged between 50 and 80 years, were included in the present study. No statistically significant difference was found between the two groups in terms of age, weight, height, gender, ASA status, operation period, or block success rate (*p* > 0.05) (Table 1).  Table 2 illustrates changes in perioperative mean arterial pressure (MAP) between the two groups. The intraoperative 5th, 10th, 20th, 30th, 60th, and 90th and intraoperative 150th minute MAP values of patients in the PCS group were found to be significantly higher than those of the USA group.  Table 3 illustrates perioperative heart rates (HR) of groups. The intraoperative 5th, 10th, 20th, 25th, 45th, 60th, 90th, and 150th minute values of patients in the PCS group were significantly higher than those in the USA group.  Table 4 illustrates the sensory and motor block beginning times, surgery beginning times, total sensory and motor block periods, time of first analgesic need, and Ramsay sedation score. The mean sensory and motor block beginning times, surgery beginning time, sensory block period time, motor block period time, and time of first analgesic need were significantly longer in the PCS group compared to those in the USA group (*p* = 0.001). The Ramsay sedation scores were similar between the groups.

Table 5 illustrates the postoperative visual analogue scale (VAS) values of the groups. The postoperative 3th hour, 6th hour, and 12th hour VAS mean values of patients in the PCS group were significantly lower than those in the USA group. In the USA group, after the 6th hour postoperatively, all the cases were in need of additional analgesic. A total of 1500 mg tramadol hcl was used for group USA. Group PCS also required additional analgesics in only 3 cases in total. A total of 300 mg of tramadol hcl was consumed for group PCS. Therefore, patients with peripheral nerve blocks (group PCS) have had a very long, painless postoperative period.

## 4. Discussion

Morbidity is significantly reduced during the perioperative period in terms of deep vein thrombosis, pulmonary embolism, transfusion need, pneumonia, respiration depression, myocardial infarction, and renal insufficiency in patients to whom peripheral nerve block is applied instead of GA [4, 5]. Additionally, peripheral blocks have many advantages compared with GA among patients with high cardiac risks; they reduce the workload of the heart, improve pulmonary function, increase oxygenation, increase mobilisation, protect cognitive functions, and reduce pain and stress [6, 7]. Naja et al. compared combined sciatic-paravertebral block with GA related to hip fracture surgery among elderly patients and reported lower hypotension incidences and fewer postoperative intensive care needs during surgery in patients to whom combined sciatic-paravertebral block was applied [8]. Their results are compatible with those of the present study. Perioperative and postoperative haemodynamic instability were not observed even among elderly patients with limited cardiac reserves in the PCS group, and none of the patients needed intensive care. Hemodynamic effects of spinal anaesthesia are based on the sympathetic block induced by anaesthesia, preoperative cardiac performance, and intravascular low volume of the patient [9–12]. Bradycardia and hypotension were observed in three patients in the USA group during the perioperative period in our clinical study.

In our study, regarding the comparisons of repeated measurements of the haemodynamic parameters between groups mean arterial pressure (MAP), heart rate (HR) values were found to be statistically significant in the USA group compared with that in the PCS group. However, the differences were not clinically significant. Lower VAS scores in the early postoperative period and a longer time of first analgesic need reduced analgesia demand and decreased the total amount of medications consumed in the postoperative period. We consider that these results provide a significant advantage for peripheral nerve block. Likewise, in a retrospective study conducted by Williams et al., femoral-sciatic nerve block applications and other anaesthesia applications were compared in 1,200 patients who underwent ambulatory orthopaedic lower extremity surgery, and patients with no block application were at a much higher risk in terms of pain. In the present study, the significance of peripheral nerve blocks in orthopaedic surgery has been emphasised [13].

Höhener et al. reported that sedated patients tolerated regional anaesthesia better, and the patient's comfort was increased during the surgical procedure [14]. In our study, patients in both groups tolerated the block comfortably because sedation was performed before the procedure, and the Ramsay sedation scores of our patients were similar.

When examining studies evaluating motor block times in peripheral nerve blocks, Adali et al. found that the motor block time was significantly longer with PCB than with SA [15]. In our study, the motor block times in the PCS group were significantly higher, at an advanced level, compared with the USA group. The results of our study are in accordance with those in the literature. In a study conducted by Mansour, comparing femoral nerve block with SA, the average surgery beginning time was 20 ± 5 minutes in the femoral group and 15 ± 5 minutes in the spinal group, and a statistically significant difference was determined [16]. In our study, the time from the completion of the patient's block procedure until the beginning of the surgical intervention was defined as the surgery beginning time. This time was significantly longer in the PCS group than in the USA group. Our results are similar to the data in the literature.

When examining studies evaluating sensory block times using peripheral nerve blocks, Urbanek et al. performed the “three in one” block procedure and found the average sensory block time to be 1,053 minutes in a 0.5% bupivacaine group, 1001 minutes in a 0.5% levobupivacaine group, and 707 minutes in a 0.25% levobupivacaine group [17]. In our study, the sensory block duration was determined to be statistically significantly longer in the PCS group than in the USA group. Our results were found to be consistent with the data in the literature.

As a result of slow injection of local anaesthetics and its use in small volumes, unilateral spinal anaesthesia ensures decrease in sympathetic block due to less spinal segment involvement, and recovery is rapid [18]. Studies in which hyperbaric bupivacaine was used recommend that patients lie over for 10 or 20 min to intensify the block on the operation side for unilateral spinal anaesthesia [19, 20]. Unilateral spinal anaesthesia was selected in this study because of the superiority of haemodynamic stability.

In their study, Aksoy et al. compared PCS block combined with sciatic nerve block and continuous spinal anaesthesia (CSA) using USG in hip arthroplasty; in terms of the perioperative results, these authors obtained significantly more stable haemodynamic data in the PCS block group than in the CSA group. In terms of the postoperative results, the time of first analgesic need was extended [21]. The results of the study of Aksoy et al. are in agreement with those of our study. Demirel et al. compared patients undergoing PCB+sciatic block combined with L1 paravertebral block with patients undergoing unilateral single-dose spinal anaesthesia in partial hip arthroplasty surgery. These authors determined that although there was no significant difference between the two groups in terms of perioperative haemodynamic stability, the time of first analgesic need in the L1 paravertebral block and PCB+sciatic block group was longer during the postoperative period [22]. While the postoperative results of Demirel et al. are compatible with those of our study, our perioperative results are not compatible because perioperative haemodynamic stability was increased in the PCS group.

In studies conducted by MacFarlane et al. and Wedel and Horlocker, it was determined that block failure varied between 0% and 67% in peripheral nerve blocks; however, this was closely related to the experience and skills of the person performing the block and localisation of the nerve where the block was applied [11, 12]. We used regional anaesthesia techniques more frequently than GA for lower extremity surgery at our clinic. Therefore, block failure was not observed among all of the patients included in this study.

In conclusion, sciatic nerve block combined with the PCB technique can be used safely, as indexed by the efficacy and side-effect profile, in total knee arthroplasty for elderly high-risk patients. In the literature, peripheral nerve blocks have been used more in hip arthroplasty. However, high-risk patients are frequently encountered in orthopaedic knee surgery. Peripheral nerve blocks should be used more in clinical practice in orthopaedic surgery.

Moreover, because of the advantages of a long duration of postoperative analgesia and low requirement for additional analgesia, this method combined with peripheral nerve block technique can be considered a good option for elderly high-risk patients.

## Figures and Tables

**Table 1:** Demographic data (mean ± SD).

	Group USA (*n* = 30)	Group PCS (*n* = 30)	*p* value
Age (year)	49.13 ± 13.25	58.5 ± 12.66	0.33
Weight (kg)	71.66 ± 10.2	74.44 ± 9.17	0,42
Height (cm)	166.18 ± 32.10	169.55 ± 17.88	0.50
Gender (F/M)	20/10	24/6	0.39
ASA II/III	13/17	9/21	0.31
Operation time (min)	81.19 ± 20.95	76.44 ± 17.66	0.26

**Table 2:** Intraoperative mean arterial pressure changes (mmHg) (mean ± SD). In between-groups comparisons; ^*∗*^*p* < 0.05.

Intraoperative mean arterial pressure values (mmHg) (MAP)	Group USA	Group PCS	*p* value
0th min	102.78 ± 9.16	106.38 ± 10.79	0.06
5th min	92.05 ± 1.11	101.09 ± 10.05	0.025^*∗*^
10th min	91.44 ± 8.75	*95.69 ± 9.73*	0.01^*∗*^
20th min	88.57 ± 13.11	91.93 ± 9.50	0.04^*∗*^
25th min	87.12 ± 8.37	92.06 ± 9.39	0.07
30th min	86.43 ± 12.30	89.75 ± 7.15	0.05^*∗*^
45th min	84.41 ± 8.30	89.97 ± 9.82	0.07
60th min	85.12 ± 8.00	91.28 ± 8.82	0.04^*∗*^
90th min	86.14 ± 9.23	91.75 ± 8.84	0.03^*∗*^
120th min	88.80 ± 4.87	90.68 ± 9.20	0.07
150th min	89.69 ± 4.71	99.1 ± 2.94	0.05^*∗*^

**Table 3:** Intraoperative heart rate (HR) changes. In between-groups comparisons; ^*∗*^*p* < 0.05.

Intraoperative HR values	Group USA	Group PCS	*p* value
0th min	76.93 ± 8.37	77.47 ± 8.98	0.12
5th min	70.07 ± 7.15	76.06 ± 8.52	0.028^*∗*^
10th min	70.03 ± 5.26	75.78 ± 7.82	0.045^*∗*^
20th min	69.89 ± 7.99	74.16 ± 7.58	0.022^*∗*^
25th min	67.93 ± 5.33	72.91 ± 6.49	0.04^*∗*^
30th min	68.11 ± 4.73	73.25 ± 6.63	0.12
45th min	66.82 ± 5.57	72.06 ± 6.51	0.02^*∗*^
60th min	67.21 ± 5.70	72.08 ± 6.90	0.03^*∗*^
90th min	66.00 ± 6.82	72.38 ± 8.96	0.05^*∗*^
120th min	69.62 ± 5.79	65.00 ± 8.49	0.3
150th min	76.93 ± 8.37	77.47 ± 8.98	0.041^*∗*^

**Table 4:** Anaesthesia characteristics of groups. In between-groups comparisons; ^*∗∗∗*^*p* = 0.001.

Anaesthesia characteristics of groups	Group USA	Group PCS	*p* value
Sensory block beginning time (min)	6.67 ± 2.23	21.08 ± 5.18	0.001^*∗∗∗*^
Motor block beginning time (min)	9.82 ± 3.87	28.78 ± 6.56	0.001^*∗∗∗*^
Surgery beginning time (min)	13.97 ± 2.02	25.17 ± 4.82	0.001^*∗∗∗*^
Sensory block period (min)	233.4 ± 10.2	772.2 ± 48.4	0.001^*∗∗∗*^
Motor block period (min)	191.4 ± 27.2	476.4 ± 34	0.001^*∗∗∗*^
Time of first analgesic need (min)	233.4 ± 66.6	664.8 ± 47.8	0.001^*∗∗∗*^
Ramsay sedation score	2.37 ± 0.6	2.44 ± 0.4	0.82

**Table 5:** Postoperative VAS (visual analogue scale) values (mm) (mean ± SD). In between-groups comparisons; ^*∗∗*^*p* < 0.01; ^*∗*^*p* < 0.05.

Postoperative VAS values	Group USA (mean ± SD)	Group PCS (mean ± SD)	*P* value
1th hour	0	0	0.058
2th hour	0	0	0.052
3th hour	20 ± 11	0	0.004^*∗∗*^
6th hour	30 ± 17	0	0.003^*∗∗*^
12th hour	30 ± 19	20 ± 12	0.016^*∗*^
18th hour	20 ± 21	30 ± 11	0.052
24th hour	30 ± 28	30 ± 10	0.06
